# ADA2b and GCN5 Affect Cytokinin Signaling by Modulating Histone Acetylation and Gene Expression during Root Growth of *Arabidopsis thaliana*

**DOI:** 10.3390/plants11101335

**Published:** 2022-05-18

**Authors:** Foteini Tsilimigka, Stylianos Poulios, Areti Mallioura, Konstantinos Vlachonasios

**Affiliations:** 1 Department of Botany, School of Biology, Faculty of Science, Aristotle University of Thessaloniki, 54124 Thessaloniki, Greece; ftsilimi@gmail.com (F.T.); spoulios@bio.auth.gr (S.P.); aretmall@bio.auth.gr (A.M.); 2 Postgraduate Program Studies “Applications of Biology—Biotechnology, Molecular and Microbial Analysis of Food and Products”, School of Biology, Faculty of Science, Aristotle University of Thessaloniki, 54124 Thessaloniki, Greece; 3 Natural Products Research Centre of Excellence (NatPro-AUTh), Center of Interdisciplinary Research and Innovation of Aristotle University of Thessaloniki (CIRI-AUTh), 57001 Thessaloniki, Greece

**Keywords:** ADA2a, ADA2b, columella cells, cytokinin, histone acetylation, GCN5, gene expression, root growth, IPT, auxin

## Abstract

In *Arabidopsis thaliana*, the histone acetyltransferase GCN5 and the associated coactivator ADA2b regulate root growth and affect gene expression. The cytokinin signaling reporter *TCS*::*GFP* was introduced into *gcn5-1*, *ada2b-1*, and *ada2a-2*, as well as the *ada2a-2ada2b-1* mutants. The early root growth (4 to 7 days post-germination) was analyzed using cellular and molecular approaches. TCS signal accumulated from the fourth to seventh days of root growth in the wild-type columella cells. In contrast, *ada2b-1* and *gcn5-1* and *ada2a-2ada2b-1* double mutants displayed reduced TCS expression relative to wild type. Gene expression analysis showed that genes associated with cytokinin homeostasis were downregulated in the roots of *gcn5-1* and *ada2b-1* mutants compared to wild-type plants. H3K14 acetylation was affected in the promoters of cytokinin synthesis and catabolism genes during root growth of Arabidopsis. Therefore, GCN5 and ADA2b are positive regulators of cytokinin signaling during root growth by modulating histone acetylation and the expression of genes involved in cytokinin synthesis and catabolism. Auxin application in the roots of wild-type seedlings increased *TCS*::*GFP* expression. In contrast, *ada2b* and *ada2ada2b* mutant plants do not show the auxin-induced TCS signal, suggesting that GCN5 and ADA2b are required for the auxin-induced cytokinin signaling in early root growth.

## 1. Introduction

Cytokinins (CK) are N6-substituted adenine derivatives discovered as cell division promoting factors [1]. CKs are involved in several plant developmental and growth processes, including root morphogenesis [2,3,4]. The active CKs in plants are isopentenyl adenine, trans-zeatin (tZ), and dihydrozeatin [5]. The cytokinin homeostasis is spatially and temporally regulated at synthesis and catabolism [6]. The *ATP/ADP-ISOPENTENYLTRANSFERASE* (IPT) family of genes encodes the enzymes that catalyze the rate-limiting step of cytokinin synthesis [7]. Gene expression analysis of *IPT* genes revealed cytokinins produced in different organs, including roots, at specific developmental stages [8]. The resulting isopentenyl ribotides can subsequently be converted to tZ-type cytokinins by hydroxylation of the isoprenoid side chain, catalyzed by the cytochrome P450 enzymes CYP735A1 and CYP735A2 [9]. The active forms of CKs are synthesized from the catalysis of cytokinin ribotides in a single step by the LONELY GUY (LOG) family of cytokinin nucleoside 5′-monophosphate phosphoribohydrolases [10]. The levels of bioactive CKs can also be modulated via conjugation to glucose or through the action of cytokinin oxidases (CKXs) [11]. CKXs decrease active cytokinin levels by irreversible cleaving CKs’ free-base and riboside forms [11].

Plants perceive and respond to CKs through a His-Asp phosphorelay pathway similar to the bacterial two-component system [12]. The Arabidopsis genome encodes for three cytokinin receptors, the transmembrane histidine kinases ARABIDOPSIS HIS KINASE 2 (AHK2), AHK3, and AHK4/WOL1(WOODEN LEG 1)/CRE1 (CYTOKININ RESPONSE1) [13]. Cytokinin binds the CHASE domain of the receptors, activating the cytosolic histidine-kinase domain and causing autophosphorylation on a conserved His residue. Subsequently, the phosphate group is transferred to a conserved Asp residue within the receiver domain [2]. Then, the phosphate group is transferred to the downstream histidine phosphotransferases (HP) and response regulators (RR) proteins [2]. There are two types of RRs involved in cytokinin signaling: type-B RRs and type-A RRs [2]. The Type-B RRs are essential for the initial transcriptional response to cytokinin [14]. In contrast, type-A RRs act as cytokinin signaling negative feedback regulators [15]. Therefore, the cytokinin signal forms a positive regulatory circuit that eventually leads to transcriptional changes in the nucleus [16].

In *Arabidopsis thaliana*, the root apical meristem consists of a small group of slowly dividing cells called the quiescent center (QC), which is surrounded by stem cells, together forming the stem cell niche (SCN) [17,18]. The QC cells produce non-cell-autonomous signals that prevent the surrounding cells from differentiation, thus preserving their stem cell identity [19]. The root is divided into developmental zones. In the meristematic zone (MZ), cell division produces a pool of cells that elongate and differentiate. In the elongation zone, cells lose the ability to divide and increase their length. Finally, in the differentiation zone, the cells acquire their specific characteristics and functions, such as the Casparian strip and the formation of root hairs. Between the meristematic zone and the elongation zone, there is a boundary, the transition zone (TZ), the position of which determines the meristem size [20]. The root cap consists of the columella (COL) and the lateral root cap (LRC), and its role stems to protect the epidermis and the SCN in the MZ [21]. COL cells are organized in cell files with large sizes departing from the columella stem cells (CSCs). Moreover, COL cells are characterized by a high turnover rate that maintains a constant number of cells. As a result, COL cells couples cell proliferation, differentiation, and detachment [22,23].

CKs have been shown to act as root growth inhibitors by promoting cell differentiation in the root apical meristem [24,25,26] and affecting root branching [27]. Moreover, CKs are highly accumulated in COL cells [28]. The root meristem size and the root growth are affected by the rate of cell division in the division zone and the rate of cell differentiation at the TZ [3]. Cytokinins determine root meristem size by regulating cell differentiation at the vascular tissue [29]. The effects of cytokinin in the root meristem depend on their antagonistic interaction with the hormone auxin [3]. Cytokinins mediate cell differentiation by acting as auxin signaling and transport suppressors, thus regulating root meristem size and growth [30].

Considering possible regulation at the genomic level, eukaryotic DNA is packaged in the nucleus with the aid of histones, forming chromatin. The structural unit of chromatin is the nucleosome, a complex consisting of eight core histone proteins, two copies of each of H2A, H2B, H3, and H4, and 147 base pairs of DNA wrapped twice around the histone core [31]. Chromatin is not an inert structure but rather a dynamic one, changing in response to endogenous and exogenous signals reaching the nucleus. One of the significant ways the cell can regulate chromatin structure, and thus, all DNA-related processes, is by histone modifications [32]. Histone acetylation is a well-studied post-translational histone modification in which acetyl-groups are incorporated in the lysine residues of the amino-terminal tails of core histones [32]. It is generally linked to the activation of gene expression; the acetyl group neutralizes the positively charged lysine residue, potentially destabilizing DNA–histone interaction and relaxing chromatin structure [32].

Two enzymes control histone acetylation, histone acetyltransferases (HATs), which catalyze the transfer of acetyl-groups to histones, and histone deacetylases (HDACs), which catalyze the removal of acetyl-groups from lysine residues. The GENERAL CONTROL NON DEREPRESSIBLE 5 (GCN5) is a HAT first identified in yeast [33]. GCN5 was identified biochemically as the first histone acetyltransferase linked to transcription [34] with specificity for histone H3 lysine 14 (H3K14) [35]. GCN5 acetylates additional histone lysine residues, such as H3K9, H3K18, H3K23, H3K27, H3K36, and other histones, such as H4 and H2B. In *Arabidopsis thaliana*, GCN5 is involved in the acetylation of histone H3 at lysine 14, but it also influences acetylation in other lysine residues, such as lysine 9 and 27 [36,37,38,39]. GCN5 works in larger multiprotein complexes, such as the SAGA complex in yeast and its plant counterpart [40,41,42], where its activity is enhanced and its specificity defined. In *Arabidopsis thaliana*, there are two functional *ALTERATION/DEFICIENCY IN ACTIVATION 2 (ADA2)* genes designated *ADA2a* and *ADA2b* [43]. GCN5 physically interacts with the transcriptional adaptors ADA2a and ADA2b, enhancing its acetylation activity [43,44,45]. GCN5 (also known as HAG1) is involved in many developmental functions and abiotic and biotic stress [41,46]. Mutations in the *GCN5* gene result in pleiotropic phenotypes, including reduced growth, loss of apical dominance, serrated leaves, and aberrant root development [47,48,49]. Mutations in *ADA2b* exhibit pleiotropic developmental abnormalities resembling *gcn5-1* [47,50]. Differences in the phenotypes of *ada2b-1* and *gcn5-1* mutants suggest that ADA2b and GCN5 have both shared and distinct functions [47]. In contrast, *ada2a* mutations do not affect plant growth and development [51]. However, the double mutant *ada2aada2b* resembles more the *gcn5* phenotypes than the *ada2b*, suggesting that GCN5 functions with both ADA2a and ADA2b proteins [51]. In Arabidopsis, GCN5 and ADA2b regulate stem cell niche maintenance and proliferation by attenuating the gradient expression of PLEPHORA transcription factors [49]. In rice, the WUSCHEL-related homeobox protein WOX11 recruits the ADA2-GCN5 module to activate downstream target genes in the crown root meristem [52]. Furthermore, GCN5 targets several root meristem gene loci, specifically in developing calli, by increasing histone acetylation in their loci to reprogram and activate their expression [53]. Moreover, in Arabidopsis, GCN5 is essential for de novo shoot formation by regulating pluripotency-inducing transcription factors [53]. GCN5 and ADA2b affect cytokinin responses during gynoecium development [54] and ethylene and auxin responses throughout the plant life cycle [54,55].

In this study, we analyze the roles of ADA2b and GCN5 in the cytokinin signaling during the early root growth of Arabidopsis seedlings using genetic and molecular approaches. We observed that GCN5 and ADA2b affect the expression of genes involved in the early steps of cytokinin biosynthesis and catabolism by modulating histone acetylation in this developmental stage.

## 2. Results

### 2.1. The Transcriptional Adaptor ADA2b Affects Cytokinin Signaling during Root Growth of Arabidopsis

Cytokinin plays an essential role in root growth by promoting cell differentiation [6]. Arabidopsis plants with mutations in the *ADA2b* gene have many developmental abnormalities, including root growth and development [47,49]. More specifically, *ada2b* mutants have a shorter root compared to the wild type. *ada2b* mutants also have a shorter root than *gcn5* mutants, which is the result of a smaller mature cell size and a smaller meristem zone [47,49]. The effect of ADA2b on cytokinin’s role during the early days of root growth was evaluated by analyzing growth spatially and temporally in wild type and *ada2b-1* mutants plants carrying the *TCS*::*GFP* transgene. TCS is a synthetic promoter with cytokinin response elements [56].

**Figure 1:**
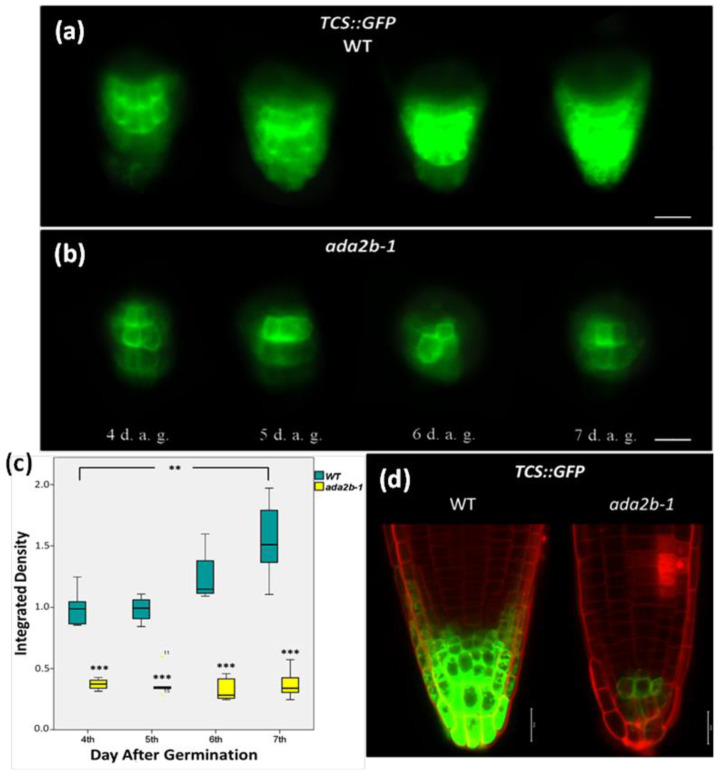
ADA2b affects cytokinin signaling at the first stages of root growth in *Arabidopsis thaliana*. The expression of reporter gene *TCS*::*GFP* in roots of (**a**) wild type (WT) and (**b**) *ada2b-1* mutant plants 4, 5, 6, and 7 days after germination (d.a.g.). Scale bars represent 50 µm. (**c**) The graph indicates the fluctuation of fluorescence density between WT and *ada2b-1* during root growth. The bars represent the range of the two quadrants, the horizontal line in the bar the median, while the terminals the minimum and maximum value of the data. Asterisks above bars of *ada2b-1* indicate statistical significance compared to the same d.a.g. of WT, while asterisks between brackets indicate the difference between 4th and 7th d.a.g., using an independent samples *t*-test: * *p* < 0.05, ** *p* < 0.01, and *** *p* < 0.001. (**d**) *TCS*::*GFP* expression in 7-day-old roots of WT and *ada2b-1* stained with iodide propidium is detected using confocal fluorescence microscopy. Scale bars represent 20 µm.

As evident by *TCS*::*GFP* expression in the wild-type plants, the cytokinin signal was detected in columella, lateral root cap, and columella initials on the fourth day of root growth after germination. The TCS signal constantly increased during and until the seventh day of root growth (Figure 1a). The cytokinin signal was accumulated at the maximum level in the outer columella cells (Figure 1a,d). However, in *ada2b-1* roots, the *TCS*::*GFP* expression was low and limited to a couple of columella cells below the QC cells, and it was absent in the lateral root cap and the outer columella cells (Figure 1b). It is worth mentioning that even in these few cells, the signal intensity was lower compared to the wild type (Figure 1d). We also quantified the fluorescence density during root growth of wild type and *ada2b-1* mutants. In the wild-type plants, the *TCS*::*GFP* signal was statistically significantly higher (two-fold) on the seventh day than on the fourth day after germination of root growth (Figure 1c). In *ada2b-1* mutants, the *TCS*::*GFP* signal remained at the same low level during the four days of growth (Figure 1c). The difference in fluorescence density between *ada2b-1* and wild type was statistically significant on all days of root growth. Overall, we conclude that ADA2b is a positive regulator of cytokinin signaling during the early days of root growth.

### 2.2. Histone Acetyltransferase GCN5 Affects Cytokinin Signaling during Root Growth of Arabidopsis

To determine if GCN5 regulates cytokinin signaling, the transgenic line *TCS*::*GFP* was crossed with *gcn5-1* mutant plants. The transgenic line *TCS*::*GFPgcn5-1* was used to study cytokinin responses in root growth in four-, five-, six-, and seven-day-old seedlings. However, we screened more than 160 *gcn5-1* seedlings by PCR-based genotyping to get only four *gcn5-1* plants with the *TCS*::*GFP* transgene. One possible explanation for the low penetrance is that in the initial *TCS*::*GFP* line, the transgene was integrated close to the *GCN5* locus. Nevertheless, we continue the observation with those lines. A *TCS*::*GFP* signal was detected throughout the lateral root cap, the columella cells, and initial columella cells with high intensity (Figure S1). Similar results were described in the literature [28,57]. In contrast, on the seventh day after seed germination, *gcn5-1* mutants displayed reduced expression of *TCS*::*GFP* transgene detected only in a few columella cells below the QC (Figure S1). Therefore, we could conclude that GCN5 acts as a positive regulator of cytokinin responses in the root growth of Arabidopsis seedlings.

**Figure 2:**
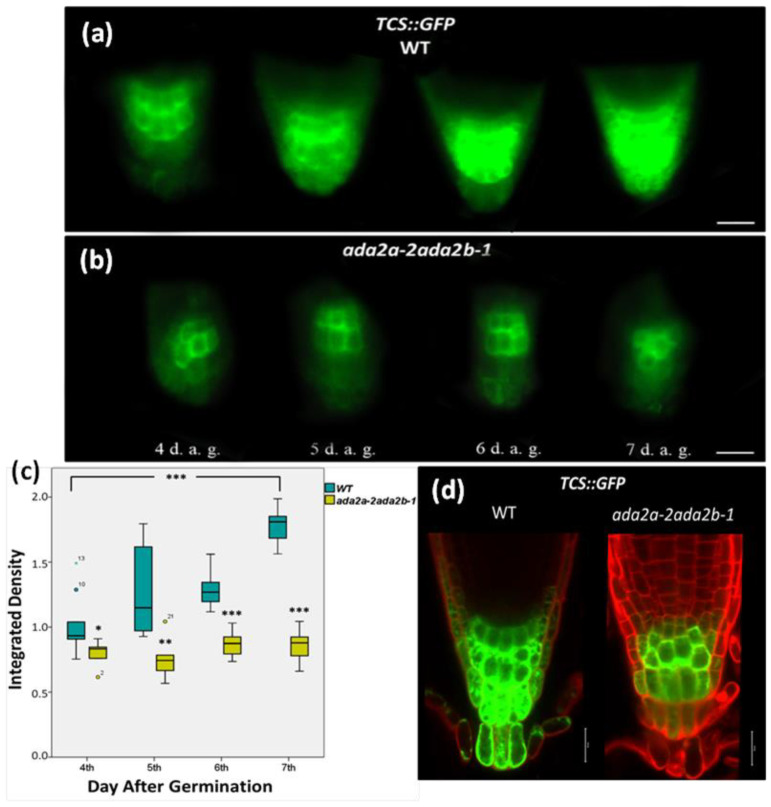
ADA2a and ADA2b affect cytokinin signaling at the first stages of root growth in *Arabidopsis thaliana*. The expression of reporter gene *TCS*::*GFP* in roots of (**a**) wild type (WT) and (**b**) *ada2a-2ada2b-1* double mutant plants 4, 5, 6, and 7 days after germination (d.a.g.). Scale bars represent 50 µm. (**c**) The graph indicates the fluctuation of fluorescence density between WT and *ada2a-2ada2b-1* during root growth. The bars represent the range of the two quadrants, the horizontal line in the bar the median, while the terminals the minimum and maximum value of the data. Asterisks above bars *ada2a-2ada2b-1* indicate statistical significance compared to the same d.a.g. of WT, while asterisks between brackets indicate the difference between 4th and 7th d.a.g., using an independent samples *t*-test: * *p* < 0.05, ** *p* < 0.01, and *** *p* < 0.001. (**d**) *TCS*::*GFP* expression in 7-day-old roots of WT and *ada2a-2ada2b-1* stained with iodide propidium is detected using confocal fluorescence microscopy. Scale bars represent 20 µm.

To overcome the obstacle with the integration of *TCS*::*GFP* close to the *GCN5* locus, we used *ada2aada2b* double mutant seedlings that phenocopy *gcn5* mutants [51]. The *ada2aada2b* double mutants resemble *gcn5* mutants in a series of phenotypes including having a larger size than *ada2b* (*gcn5* are larger than *ada2b*), displaying less pronounced leaf morphology, and having increased number of primary inflorescences, a character specific of *gcn5* but not *ada2b* mutants [47,51]. Therefore, the *TCS*::*GFP* transgenic line was crossed with the *ada2a-2ada2b-1* double mutant, and the expression of the transgene was monitored during root growth from four days to seven days after germination. The *TCS*::*GFP* expression was detected in the columella cells on the fourth day of wild-type root growth, especially in 10 cells below the QC (Figure 2a). As the root growth continued, the expression of *TCS*::*GFP* was increased in the same cells, and on the seventh day of growth, *TCS*::*GFP* was detected in high levels in all columella, the lateral root cap, and the columella initials cells (Figure 2a,d). In contrast, on the fourth day of *ada2a-2ada2b-1* root growth, the *TCS*::*GFP* expression was detected in only five columella cells and was visibly more attenuated than in the wild type (Figure 2b). As the root growth continued in *ada2a-2ada2b-1*, neither the spatial distribution nor the *TCS*::*GFP* signal intensity changed compared to the fourth day of growth (Figure 2b), as observed in the wild type (Figure 2a). Finally, on the seventh day of root growth in *ada2a-2ada2b-1*, as clearly shown in Figure 2d, the *TCS*::*GFP* signal was detected in only these five columella cells as well as with attenuated expression in the adjacent cells, including the initial lateral root cap and columella cells as well as the QC. The *TCS*::*GFP* signal was absent in outer columella cells. The difference in TCS signal between wild-type *and ada2a-2ada2b-1* was also illustrated in Figure 2c. Specifically, during the wild-type root growth, the quantity of *TCS*::*GFP* fluorescence was increased on the seventh day and was statistically significantly higher than on the fourth day (Figure 2c).

On the other hand, in the *ada2a-2ada2b-1* double mutant, the quantity of *TCS*::*GFP* fluorescence reduced per day compared to the wild type and remained constant during the root growth. Notably, the expression of the TCS signal in the *ada2a-2ada2b-1* double mutant was slightly higher than in *ada2b-1* single mutants (Figure 2c). Therefore, we can conclude that GCN5, through the associated subunits ADA2b and ADA2a, positively regulates cytokinin signaling in the Arabidopsis root.

Next, we asked if ADA2a is required for cytokinin signaling in the Arabidopsis roots. The *TCS*::*GFP* transgenic line was also crossed with the *ada2a-2* mutant, and the expression of the transgene was monitored during root growth from four days to seven days after germination. The pattern of *TCS*::*GFP* expression in the roots of the *ada2a-2* mutant was similar to the way observed in wild-type seedlings (Figure S2). *TCS*::*GFP* was detected in high levels in all columella, the lateral root cap, and the columella initials cells (Figure S2). Therefore, these results suggest that ADA2a is not required to accumulate cytokinin in the roots, indicating functional redundancy between ADA2a and ADA2b proteins.

### 2.3. GCN5 and ADA2b Regulate the Expression of Genes Involved in Cytokinin Synthesis, Catabolism, and Signaling during the Root Growth of Arabidopsis

**Figure 3:**
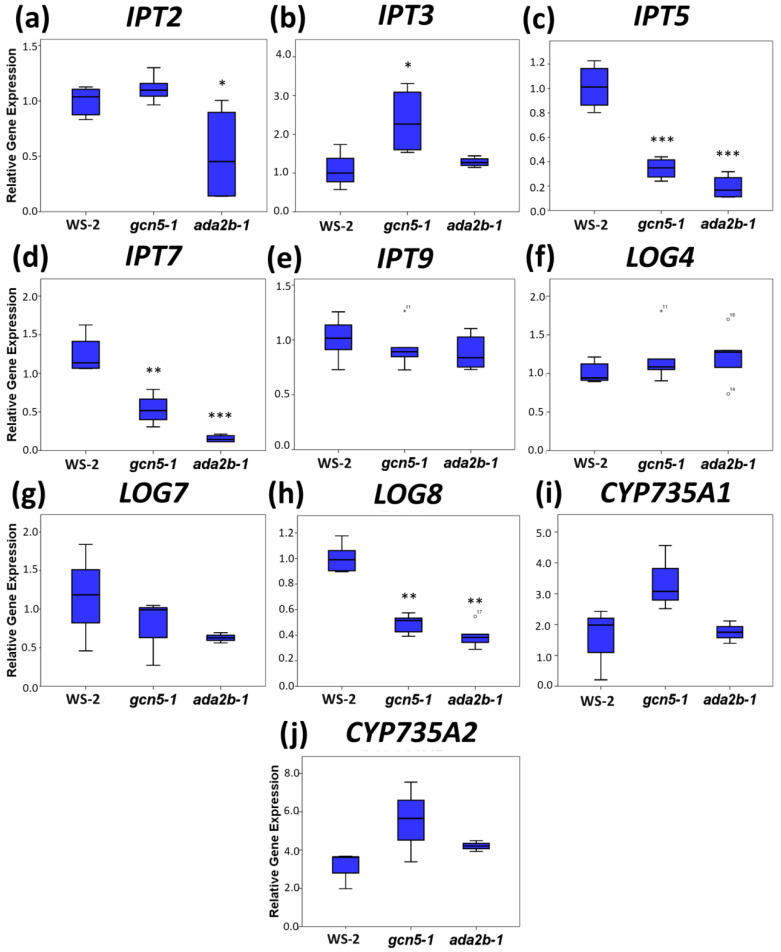
The role of GCN5 and ADA2bin cytokinin biosynthesis-related gene expression in *Arabidopsis thaliana* roots. The analysis was carried out in roots of seven-day-old Ws-2, *gcn5-1*, and *ada2b-1* plants using quantitative Reverse Transcription Polymerase Chain Reaction (qRT-PCR) for the genes (**a**) *IPT2*, (**b***) IPT3*, (**c**) *IPT5*, (**d**) *IPT7*, (**e**) *IPT9*, (**f**) *LOG4*, (**g**) *LOG7*, (**h**) *LOG8,* (**i**) *CYP735A1*, and (**j**) *CYP735A2*. The bars represent the range of the two quadrants, the horizontal line in the bar the median, while the terminals are the minimum and maximum values of the data. Asterisks indicate the statistical significance based on an independent samples *t*-test: * *p* < 0.05, ** *p* < 0.01, and *** *p* < 0.001.

To understand how GCN5 and ADA2b regulates root growth by affecting cytokinin signaling, the expression of genes involved in cytokinin homeostasis, including biosynthesis, catabolism, and signaling, was monitored in seven-day-old roots in wild type, *gcn5-1*, and *ada2b-1* mutants. The genes encoding for IPTs catalyze the first step in cytokinin biosynthesis and are expressed in a tissue and organ-specific manner throughout plant development. Specifically, *IPT3*, *IPT5*, and *IPT7* are highly expressed in the root of Arabidopsis, whereas *IPT2* and *IPT9* are ubiquitously expressed [8,58]. The *IPT2* expression is slightly downregulated only in *ada2b-1* mutants (Figure 3a). An analysis of *IPT5* and *IPT7* expression showed that both were downregulated in *gcn5-1* and *ada2b-1* roots compared to the wild type, while *IPT3* expression increased only in *gcn5-1* (Figure 3b–d). The expression of *IPT9* did not change between the mutants and the wild type (Figure 3e). These results suggest that the expression of some members of the *IPT* family is regulated by GCN5 and ADA2b. These results also indicate that GCN5 and ADA2b are positive regulators in the first steps of cytokinin biosynthesis in the roots. The next step in cytokinin biosynthesis involved the activity of *Lonely Guy (LOG)* genes that encode a cytokinin riboside 5′-monophosphate phosphoribohydrolase [59]. The expression of *LOG4* did not change between mutants and wild type, whereas *LOG7* displayed a slight but not significant reduction in the *gcn5-1* and *ada2b-1* mutants compared to the wild-type.

**Figure 4:**
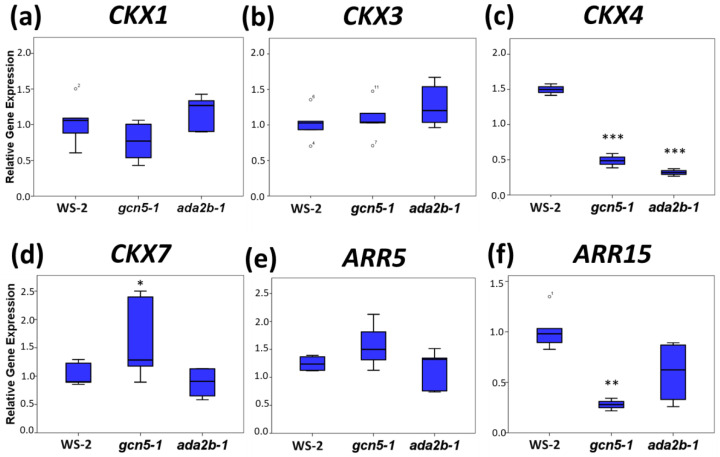
The role of GCN5 and ADA2b in cytokinin catabolism and signaling-related gene expression in *Arabidopsis thaliana* roots. The analysis was carried out in roots of seven-day-old Ws-2, *gcn5-1*, and *ada2b-1* plants using qRT-PCR for the genes (**a**) *CKX1*, (**b**) *CKX3,* (**c**) *CKX4*, (**d**) *CKX7*, (**e**) *ARR5*, and (**f**) *ARR15*. The bars represent the range of the two quadrants, the horizontal line in the bar the median, while the terminals are the minimum and maximum values of the data. Asterisks indicate the statistical significance based on an independent samples *t*-test: * *p* < 0.05, ** *p* < 0.01, and *** *p* < 0.001.

Most importantly, *LOG8* significantly reduced its expression in *gcn5-1* and *ada2b-1* mutants (Figure 3f–h). The next step in cytokinin biosynthesis involved two cytochrome P450 monooxygenases encoded by the *CYP735A1* and *CYP735A2* genes in Arabidopsis, which catalyze the trans-hydroxylation step in vitro [9]. The expression of both genes was upregulated in *gcn5-1* roots compared to wild-type and *ada2b-1*; however, these changes were not statistically significant (Figure 3i,j). In parallel, we also monitored the expression of various *CKX* family genes, which catalyze the irreversible degradation of cytokinins and are responsible for most metabolic cytokinin inactivation [6]. The expression of *CKX1*, *CKX3*, and *CKX7* was not affected in the mutants compared to the wild type (Figure 4a,b,d), but the expression of *CKX4* was significantly reduced in both *gcn5-1* and *ada2b-1* mutants (Figure 4c). These results indicate that GCN5 and ADA2b affect the cytokinin degradation pathway in the roots. Finally, we monitored the expression of some *type-A ARR* genes, negative regulators of cytokinin signaling [60], in the roots. The gene expression of *ARR15* is reduced significantly in the *gcn5-1* roots relative to the wild-type and *ada2b-1*, while no change was observed in the *ARR5* expression levels (Figure 4e,f).

### 2.4. GCN5 Promotes H3K14 Acetylation in the Loci of IPT5, IPT7, and CKX4 Genes

**Figure 5:**
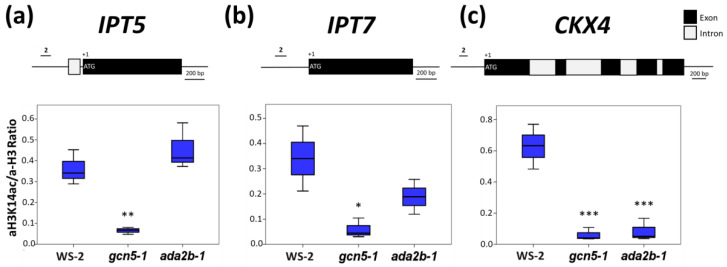
Histone acetylation of H3K14 in roots of seven-day-old Ws-2, *gcn5-1*, and *ada2b-1* plants. The promoter region was analyzed for each genomic region as indicated by the line above the gene model. (**a**) The *IPT5*, (**b**) *IPT7*, and (**c**) *CKX4* proximal promoter regions were analyzed. The immunoprecipitated DNA fragments were analyzed by qRT-PCR, and the values were obtained as a percentage of input. Antibodies against Histone H3 and acetylated Histone H3K14 were used. The ratio of H3K14ac to H3 is presented. The bars represent the range of the two quadrants, the horizontal line in the bar the median, while the terminals are the minimum and maximum values of the data. Asterisks indicate the statistical significance of three technical repeats of two mutants compared to wild type based on an independent samples *t*-test: * *p* < 0.05, ** *p* < 0.01, and *** *p* < 0.001.

Next, we examined whether the observed changes in cytokinin-related gene expression in the *gcn5* and *ada2b* single mutants resulted from changes in the acetylation status of their locus. We performed ChIP analysis in the roots of seven-day-old seedlings using antibodies for histone H3 lysine 14 acetylation (H3K14) normalized to H3 antibody. H3K14 is the GCN5 target for acetylation [36,37]. At the *IPT5* locus, H3K14 acetylation was decreased by almost three-fold in *gcn5-1* mutants in the proximal promoter region, the distal promoter, and the 3′UTR regions. However, this was not observed in the *ada2b-1* mutants. This result suggests that in this locus, H3K14 acetylation is regulated by GCN5 action (Figure 5a and Figure S3a). Furthermore, ADA2b functions differentially from GCN5 in H3K14 acetylation in the *IPT5* gene.

We also analyzed the histone H3K14 acetylation status in the proximal promoter region of *IPT7* and *CKX4*. In the promoter regions of *IPT7* (−160 to −70 from TSS) and *CKX4* (−135 to −34 from TSS) loci, H3K14 acetylation was reduced significantly in the roots of *gcn5-1* mutants (Figure 5b,c). Interestingly, H3K14 acetylation was also decreased in *ada2b-1* in the *CKX4* promoter region (Figure 5c). In the *IPT7* promoter region, the H3K14 acetylation was also reduced in the *ada2b-1* roots but was not statistically significant (Figure 5b). The H3K14 acetylation levels in the *CYP375A1* promoter region were used as a negative control, since the expression of this gene has no significant change. As such, the H3K14 acetylation levels do not show a statistically significant difference in the proximal promoter of the gene in *gcn5-1* and *ada2b-1* mutants compared to wild-type roots (Figure S3b).

### 2.5. GCN5 and ADA2b Is Required for the Auxin-Induced Cytokinin Signaling in Arabidopsis thaliana Root

**Figure 6:**
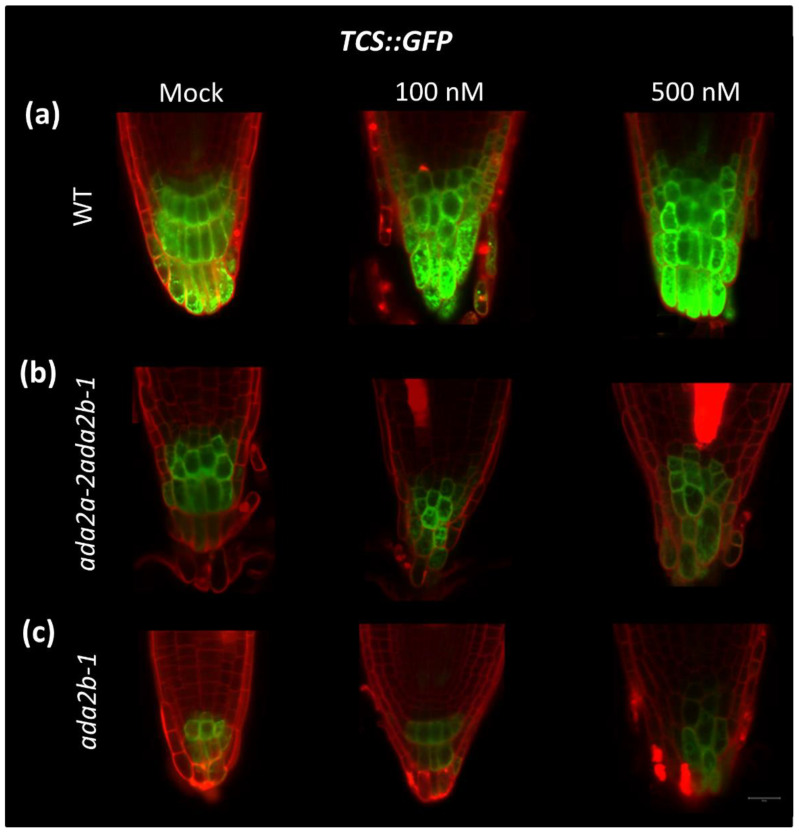
The role of ADA2b and ADA2a in auxin-induced cytokinin signaling during early root growth in *Arabidopsis thaliana*. The expression of reporter gene *TCS*::*GFP* in roots of (**a**) wild type (WT), (**b**) *ada2a-2ada2b-1* double mutant, and (**c**) *ada2b-1* mutant treated with 100 or 500 nM exogenous auxin IAA for 24 h or grown in control media (mock). Roots stained with iodide propidium (PI: 10 μg/mL) and observed on confocal fluorescence microscopy. The scale bar represents 20 μm.

Cytokinin affects the differentiation of root cells by antagonizing the auxin-induced cell division [30]. To further understand the role of GCN5 and ADA2b in regulating the cytokinin signaling pathway in Arabidopsis root, we applied IAA (Indole-3-Acetic Acid) for 24 h at two different concentrations (100 and 500 nM) in wild-type plants, *ada2b-1* and *ada2a-2ada2b-1* double mutants that carried the *TCS*::*GFP* transgene. The spatial distribution of the *TCS*::*GFP* signal was not different between the mock-treated plants and the IAA-treated plants (Figure 6a). However, the application of 100 nM IAA to wild-type plants caused an increase in the intensity of the TCS signal, especially in the outer columella cells. Furthermore, treatment with 500 nM IAA resulted in even higher levels of *TCS*::*GFP* expression in the same cells. These results indicate an auxin-induced cytokinin accumulation in the columella cells.

In contrast, in *ada2a-2ada2b-1*, which represents the GCN5-impaired function, the intensity of the TCS signal was not altered after application of either 100 or 500 nM IAA (Figure 6b). The same pattern was also observed in *ada2b-1*, where the expression pattern and signal strength did not change after treatment with both IAA concentrations (Figure 6c). These results indicate that the transcriptional adaptor ADA2b and the histone acetyltransferase GCN5 are required for the auxin-induced cytokinin signaling in the root columella cells.

## 3. Discussion

Herein, we provide evidence that the histone acetyltransferase GCN5 and the associated transcriptional adaptor ADA2b affect cytokinin signaling during the early stages of root growth in Arabidopsis seedlings using the cytokinin sensitive reporter *TCS*::*GFP*, designed to approximate global ARR-B transcript levels [61]. We confirmed previous observations that *TCS*::*GFP* expression was detected in the root cap, especially in the columella cells, lateral root cap, and columella initials [61,62]. The *TCS* expression was increased during the first days of root growth, indicating that cytokinin signaling is involved in the early root growth events. The TCS signal could reflect bioactive cytokinin levels or the TCS output results from variations in the abundance of downstream signaling components [62]. The number of differentiated COL layers is reduced in *ada2b* and *ada2aada2b* double mutants suggesting that both ADA2a and ADA2b are required for the proliferation of these cells, confirming previously reported results [49]. In the *gcn5* mutants, this phenotype arose from smaller meristem cells [63]. The location of cytokinin maximum as monitored by *TCS*::*GFP* was affected in *ada2b* and *ada2aada2b* mutant plants. The cytokinin levels in the columella cells were also reduced. Therefore, GCN5 and ADA2b positively regulate TCS signals in the root cap during early root growth stages. As a result, we asked if GCN5 and ADA2b affect the expression of CK biosynthetic genes. Indeed, GCN5 and ADA2b positively regulate the expression of genes involved in cytokinin biosynthesis, especially *IPT5*, *IPT7*, and *LOG8*. Both *IPT5* and *IPT7* genes are highly expressed in the *pWOL*::*GFP* transgenic line, a marker for stele cells [64]. Using single-cell RNA-seq approaches, *IPT7* was identified as a potential cell type-specific marker for the metaxylem [65]. The quadruple mutants *ipt1ipt3ipt5ipt7* almost completely depleted endogenous cytokinin levels due to deficient biosynthesis show substantial growth retardation both of the shoot and root [66]. In the CSCs, LRC, and the differentiated COL cells, the isopentenyl adenine, cis-zeatin (cZ), and trans-zeatin (tZ); and its conjugates are accumulated at very high levels [28]. Accordingly, several genes that participate in CK homeostasis are also expressed in COL and LRC [6,20,28,67].

In the *M0028*::*GFP* line [68], a specific marker for gene expression in LRC, COL, CSCs, and QC cells, the cytokinin oxidase gene *CKX4* was accumulated [6,20]. CKX4 is also a potential cell type-specific marker for columella cells [65]. Our results suggest that *CKX4* is regulated by both ADA2b and GCN5 action by modulating H3K14 acetylation in its promoter.

Diminishing levels of cytokinins lead to a larger MZ and a delay in the onset of endoreplication, as exemplified by the triple mutant *ipt3ipt5ipt7* or by causing an increase in CK catabolism by the overexpression of CKX genes [6,14,29,69]. GCN5 and ADA2b are required for the root meristematic zone by regulating the PLETHORA (PLT) pathway [49]. GCN5 regulates stem cell niche maintenance independent from ADA2b by not acting on similar targets in stem cells and stem cell daughters [49]. We found that GCN5 but not ADA2b affected the H3K14 acetylation levels at *IPT5* and *IPT7* promoters, even though both genes are expressed at lower levels in *gcn5* and *ada2b* mutants. Therefore, GCN5-dependent histone acetylation is required to express cytokinin biosynthesis and cytokinin degradation pathways.

CK and auxin crosstalk are often known to regulate the MZ size by controlling the transition from proliferation to elongation [70]. We found that IAA treatment promotes cytokinin signaling in the wild-type root cap cells, suggesting an auxin-induced cytokinin response. Indeed, exogenous auxin treatment was reported to upregulate *IPT5* and *IPT7* expression in roots [8]. Furthermore, the expression of the B-types *ARR1* and *ARR12* was rapidly induced by auxin treatment in the transition zone area [70,71]. Herein, we showed that GCN5 (the action of both ADA2a and ADA2b) and ADA2b are required for the auxin-induced B-type ARR signaling, as indicated by the TCS signal, in the LRC, COL, and CSCs cells. GCN5 is also known to affect cytokinin signaling in Arabidopsis gynoecium [54]. Similarly, auxin application increased the TCS signal in stage 10 Arabidopsis gynoecium, particularly in presumptive provasculature cells and septa primordia [72]. Furthermore, ADA2b (also known as PROPORZ1) and GCN5 mediate cytokinin signals in the control of cell proliferation [50,53]. GCN5 reprograms the epigenetic status of several root-meristem genes through histone acetylation and activates their transcription on auxin-rich callus induction media (CIM) [53]. The control of cell proliferation and cell differentiation of the COL is maintained by the action of WUSCHEL-RELATED HOMEOBOX 5 (WOX5), a homeodomain transcription factor expressed in the QC cells. GCN5 triggers acetylation in the promoter of the WOX5, SCARECROW (SCR), PLT1, and PLT2 in the root meristem [53]. Furthermore, several transcription factors interact with ADA2b to recruit the GCN5-histone acetylation machinery to specific auxin-responsive genes [73,74].

Five days after germination, maximal meristem size and stabilization of the transition zone are established by the auxin/PLT/ARR-B network [70]. Our result suggests a synergistic network of ARR-B signaling and auxin-regulated by GCN5 and ADA2b complex that modulate histone acetylation of cytokinin-related genes in the root cap during the early days of root growth. GCN5-ADA2b could affect cytokinin levels by regulating cytokinin biosynthesis and catabolism in this model. Furthermore, auxin-induced cytokinin signaling is also dependent on GCN5-ADA2b activity. Our work indicates a crucial role of GCN5-dependent histone acetylation in the interplay between cytokinin signaling and regulatory networks during root growth. Root meristem size constantly changes in response to external and internal conditions, signaled by the changing levels of histone acetylation and other chromatin modifications to modulate hormone responses, such as auxin and cytokinin. Thus, the next challenge is to understand how roots continually integrate all this information to alter root growth dynamically and how this can be coordinated across the whole root system.

## 4. Materials and Methods

### 4.1. Plant Materials and Growth

The *Arabidopsis thaliana* (L) Heynh. *Gcn5-1*, *ada2a-2*, *ada2b-1*, and *ada2a-2ada2b-1* were previously described in [47,51]. The transgenic line *TCS*::*GFP* [56] was obtained by the Nottingham Arabidopsis Stock Centre (NASC). Seeds were sterilized and cold-treated at 4 °C for 3–4 days in the dark. For plating, seeds were sown on Gamborg B5 (GB5) medium (Duchefa Biochemie, Amsterdam, the Netherlands) supplemented with 1% sucrose (Duchefa) and 0.8% phytoagar (Duchefa) and adjusted at pH 5.6–5.8. Plants were grown at 20–22 °C with 100–150 μmol m^−2^ s^−1^ cool-white fluorescent lamps under long-day conditions (16 h light/8 h dark). Commercially available soil, Terrahum^®^ (Deutsche Kompost Handelsgesellschaft), was used for cultivation. Soil-grown plants were irrigated twice weekly with water. For hormone treatment, 7-day-old plants were transferred from solid GB5 medium to liquid GB5 medium supplemented with 100 nM or 500 nM of IAA (3-Indoleacetic acid, Sigma-Aldrich, St Louis, MO, USA), which was dissolved in ethanol. The control seedlings were mock-treated with ethanol.

### 4.2. Genetic Analysis and Genotyping

The *TCS*::*GFPgcn5-1* mutant was previously described by [54]. The *TCS*::*GFPada2b-1/+*, *TCS*::*GFPada2a-2/+*, and *TCS*::*GFPada2a-2ada2b-1/+* plants were created by crossing the *TCS*::*GFP* line with the *ada2a-2ada2b-1/+* heterozygous plants. The F1 was left to self-fertilize, and desired genotypes were identified in the F2 and F3 generations. PCR tracked the required genotypes with specific primers (Supplemental Table S1). When applicable, kanamycin resistance of the *ada2b-1* allele was used to facilitate the selection.

### 4.3. Gene Expression Analysis

Roots of 7-day-old WT, *gcn5-1*, and *ada2b-1* seedlings were collected and flash-frozen in liquid nitrogen. The selection of homozygous *ada2b-1* was phenotypically made based on their longer hypocotyl and shorter root compared to WT [47]. Total RNA was extracted according to the Nucleospin^®^ RNA Plant kit (Macherey-Nagel, Duren, Germany). Reverse transcription was performed in at least three biological repeats using 0.5 μg total RNA based on PrimeScript™ first strand cDNA Synthesis Kit (TaKaRa, Shiga, Japan). Quantitative reverse-transcription polymerase chain reactions (RT-qPCRs) were performed by using the AMPLIFYME SG Universal Mix (AM02) (BLIRT SA, Gdańsk, Poland) at the ABI StepOne™ system (Applied Biosystems, Foster City, CA, USA). Each sample was analyzed in triplicate, and the *At4G26410* expression was used as a reference (Supplemental Table S1). Data were analyzed according to the ΔΔCt method using StepOne Software ν2.1. Statistical significance was calculated using a one-way ANOVA, with Fisher’s Least Significant Difference (LSD) and Post Hoc Test with a 95% confidence interval using IBM SPSS Statistics software 23.0 (Statistical Product and Service Solutions), USA.

### 4.4. Microscopy

At least 30 roots of 4-, 5-, and 6-day-old and 80 7-day-old seedlings were observed in a Zeiss AxioImager.Z2 (Carl Zeiss AG, Munich, Germany) equipped with a digital AxioCam MRc 5 camera for green fluorescent protein (GFP) detection. The integrated intensity of GFP was measured by ImageJ software, and values were normalized to background fluorescence [75]. Statistical significance was calculated using an independent samples *t*-test, with a 95% confidence interval, using IBM SPSS Statistics software 23.0 (Statistical Product and Service Solutions), USA.

Seven-day-old seedlings with or without IAA treatment were stained with 10 µg/mL propidium iodide (PI) (Sigma-Aldrich, St Louis, MO, USA) for about 5 min. Root caps were visualized under a Zeiss Observer.Z1 microscope (Carl Zeiss AG, Munich, Germany), equipped with an LSM780 confocal laser scanning module to detect GFP. Imaging was achieved with ZEN2011 software according to the manufacturer’s instructions.

### 4.5. Chromatin Immunoprecipitation (ChIP)

A ChIP assay was performed with minor modifications [45,55]. For this assay, only the roots of 7-day-old seedlings were used. In total, 250, 650, and 900 roots for wild type ecotype Ws-2, *gcn5-1*, and *ada2b-1* plants, respectively, were harvested. The *ada2b-1* roots were selected from a segregating population of approximately 4000 seedlings. The total amount of tissue used for the assay was 30 mg per sample. The antibodies against acetylated histone H3K14 (Anti-Histone H3 (Lys14), EMD Millipore #07-353) and H3 (ChIPAb + Histone H3 C-term, EMD Millipore #17-10046) were used. Immunoprecipitated DNA was diluted in water and analyzed by RT-qPCR using specific primers (Supplemental Table S1). RT-qPCRs were performed using the AMPLIFYME SG Universal Mix (AM02) (BLIRT SA, Gdańsk, Poland) at the ABI StepOne™ system (Applied Biosystems, Foster City, CA, USA). A standard curve was constructed using the input samples in five 10-fold serial dilutions. All data obtained by q-PCR were presented as a percentage of input. The value of each immunoprecipitated sample was normalized to the input. The ratio of acetylated H3K14 to H3 values of each genotype is presented. Statistical significance was calculated using a one-way ANOVA, with Fisher’s Least Significant Difference (LSD) and Post Hoc Test with a 95% confidence interval using IBM SPSS Statistics software (Statistical Product and Service Solutions), USA.

## Data Availability

The data that support the findings of this study are available from the corresponding author upon reasonable request.

## Supplementary Materials

The following supporting information can be downloaded at: https://www.mdpi.com/article/10.3390/plants11101335/s1, Figure S1: Cytokinin signaling at the first stages of *gcn5-1* root growth in *Arabidopsis thaliana*; Figure S2: The effect of ADA2a on cytokinin signaling at the first stages of root growth in *Arabidopsis thaliana*; Figure S3: Histone acetylation in additional regions of *IPT5* locus and the promoter of *CYP735A1* locus; Table S1: List of primers used in this study.
