# Supplementary material for: ADA2b and GCN5 Affect Cytokinin Signaling by Modulating Histone Acetylation and Gene Expression during Root Growth of Arabidopsis thaliana
Source: Plants (Basel). 2022 May 18;11(10):1335. doi: 10.3390/plants11101335 (PMC9148027; doi:10.3390/plants11101335)
Supplement: Supplementary file 1 [file plants-11-01335-s001.zip › plants-1698116-supplementary.pdf]

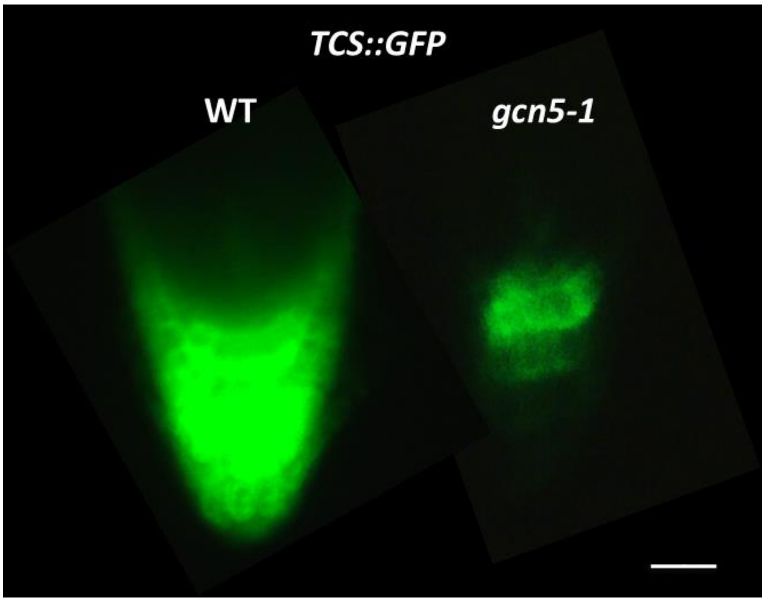

Figure S1. Cytokinin signaling at the first stages of root growth in *Arabidopsis thaliana*. The expression of reporter gene *TCS::GFP* in roots of wild type (WT) and *gcn5-1* mutant 7 days after germination (d.a.g.) is represented. Scale bars represent 50 μm.

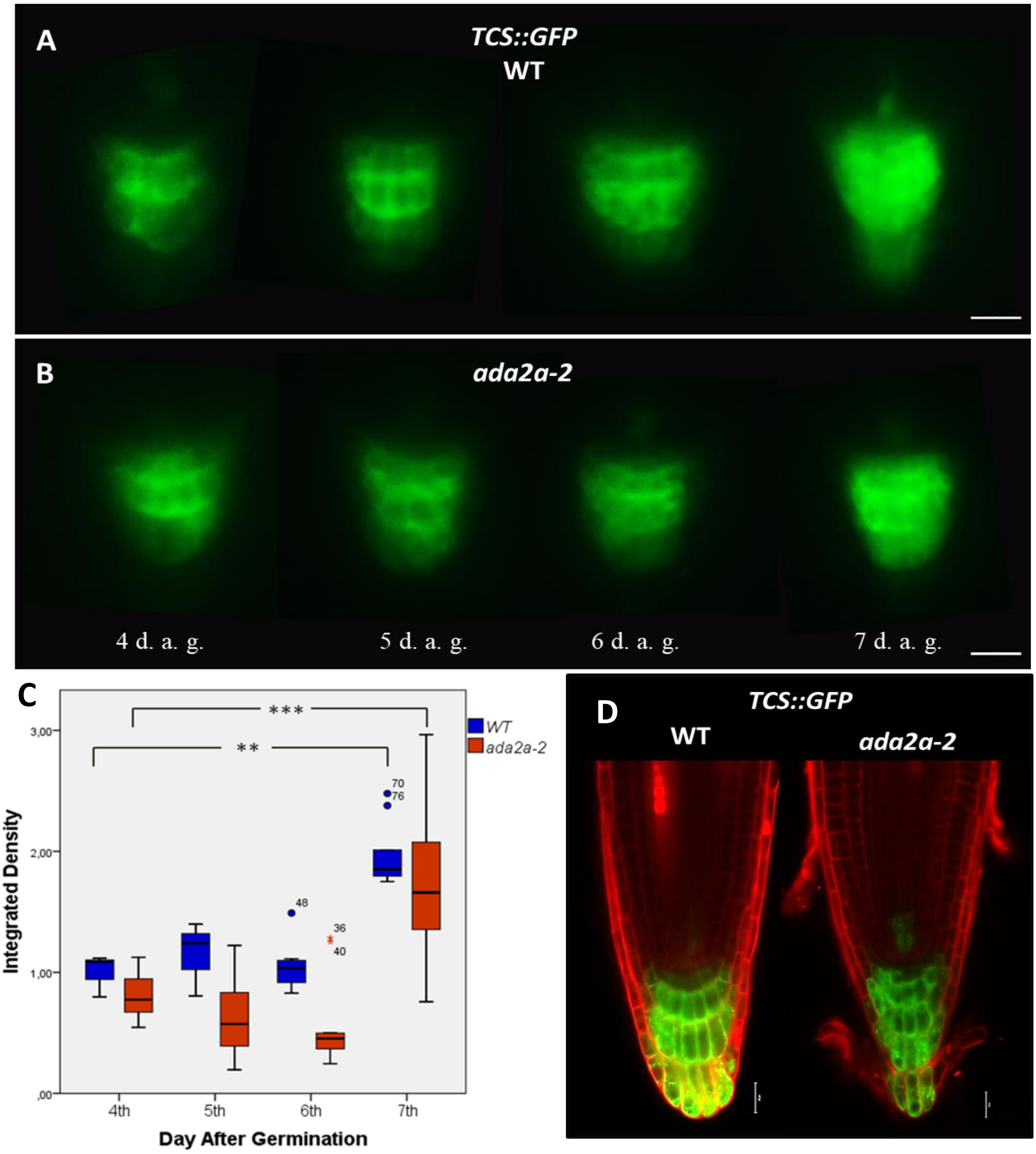

Figure S2. Cytokinin signaling at the first stages of root growth in *Arabidopsis thaliana*. The expression of reporter gene *TCS::GFP* in roots of (A) wild type (WT) and (B) *ada2a-2* mutant 4, 5, 6 and 7 days after germination (d.a.g.). Scale bars represent 50 μm. (C) The graph indicates the fluctuation of fluorescence density between WT and *ada2a-2* during root growth. The bars represent the range of the two quadrants, the horizontal line in the bar the median, while the terminals the minimum and maximum value of the data. Asterisks above bars of *ada2a-2* indicate statistical significance compared to the same d.a.g. of WT, while asterisks between brackets indicate the difference between 4th and 7th d.a.g., using Independent-Samples T test: *P < 0.05, **P < 0.01 and ***P < 0.001. (D) *TCS::GFP* expression in 7-days old roots of WT and *ada2a-2ada2b-1* stained with iodide propidium is detected using confocal fluorescence microscopy. Scale bars represent 20 μm).

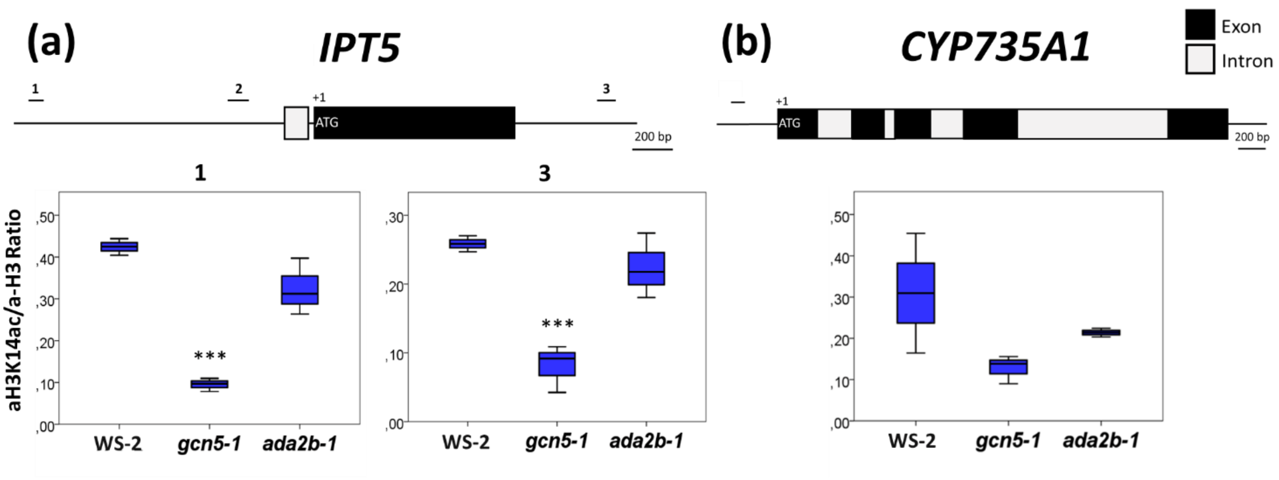

Figure S3. Histone acetylation of H3K14 in roots of seven-days old plants *Ws-2*, *gcn5-1* and *ada2b-1*. (A) The upper promoter region (1) and the downstream 3'UTR region (3) was analyzed for the *IPT5* genomic locus. The proximal promoter region is represented by (2), (B) The promoter region for the *CYP735A1* locus was analyzed. The immunoprecipitated DNA fragments were analyzed by quantitative Reverse Transcription Polymerase Chain Reaction (qRT-PCR) and the values obtained as a percentage of input. Antibodies against Histone H3 and acetylated Histone H3K14 were used. The ratio of H3K14ac to H3 is presented. The bars represent the range of the two quadrants, the horizontal line in the bar the median, while the terminals the minimum and maximum value of the data. Asterisks indicate the statistical significance of three technical repeats of two mutants compared to wild type based on Independent-Samples T test: *P < 0.05, ** P < 0.01 and ***P < 0.001.

Table S1. List of primers used in this study.

Experiment	Target Sequence	Sequence (5' → 3')
Genotyping	*GCN5*	AGTGGGGGCACACTCGTTTCAAATTATTC
		TTGAGATTTAGCACCAGATTGGAGACCTG
	gcn5-1	CATTTTATAATAACGCTGCGGACATCTAC
		TTGAGATTTAGCACCAGATTGGAGACCTG
	ADA2b	ACTCCTCACAAATGTGATCACCCATACCG
		CTCCATCTCCGCCAAGAGTTGCTCAG
	ada2b-1	CATTTTATAATAACGCTGCGGACATCTAC
		CTCCATCTCCGCCAAGAGTTGCTCAG
	ADA2a	CTAGCTTCTCGTCCTGCTGAGG
		TGAGTCACCTATTCCCTTATGAACCAGCA
	ada2a-2	CTAGCTTCTCGTCCTGCTGAGG
		CATTTTATAATAACGCTGCGGACATCTAC
	mGFP	TCAAGGAGGACGGAAACATC
		AAAGGGCAGATTGTGTGGAC
	clv1-1	TTTTGAAGCCCATGTTGCTG
		GTTCGCCACGGATTTAGGAG
Gene Expression	*At4G26410*	GAGCTGAAGTGGCTTCCATGA
		GGTCCGACATACCCATGATCC
	ARR5	GCCTCGTATCGATAGATGTCTTGAAGAAGG
		TCTGATAAACTCAGATCTTTGCGCGT
	ARR15	CTGCTTGTAAAGTGACGACTGTTG
		AGTTCATATCCTGTTAGTCCCGGC
	CKX1	AGGCAACAGGAAGTCCTCAA
		Tagcctgagcggttacaagg
	CKX3	CGCTATACCCGAGGAAGATG
		TCAAAAGCCTCCCAATTGTC
	CKX4	TCCACCAGCTGATTGCAGTA
		ATCCCACAAACCGAAACAAA
	CKX7	TGTTTGATCCCATGGCTATACT

		aatatgaggggTCAAAGAGACC
	CYP735 A1	GCTTTACCAAGGCATTCCAA
		CCCCAATCGTTGTGCTTACT
	CYP735 A2	CAAATCCCTCATGTCACTCAA
		CCACTTGATCTATGAAGGGAGA
	LOG4	CACCTACTGCCAAGGAGCTT
		CCGCTCTATCTCCCAACAAA
	LOG7	TTTCCGCTGTACACTGCTTTT
		GGATGAAGTGGACCGGATAA
	LOG8	TTGTGGTTTCTGCTCCAACA
		CCGGGTAATCTCCGAGTTCT
	IPT2	TCGGCTAGAAACGGTCTTTG
		GGTTTAACCACTTGCGCATT
	IPT3	CAGAAACCGCGACACAGTAT
		CCAAGATGGATGCTAACGTG
	IPT5	TGTTTTACCTCACCGGGAAA
		GATGACCACCATTTGGAAGG
	IPT7	GTAAGATGCCGCCAAACAGT
		GACCAAGCGAGAGAATCGTC
	IPT9	GTTCCGGTGTGAGCCTATGT
		TCCGGTATCTCCACCATCTC
ChIP analysis	*IPT5 1*	ATGTTATGAACGTCCCGTTTC
		TGGCTTATTCCCAAACATCC
	IPT5 2	TGAGACACGTGTTATGGAGCTA
		CGCGGCTTCGAATTTATAGT
	IPT5 3	GGATCCAACGGACTAAAAAGG
		GGTCACCCCAAACACGTATC
	IPT7	GTTGTGGAATTTCAAAGGGTTT
		GCCGTGTTTTCTCTGACTTTG
	CKX4	TTTCGAAGTCAATGCAGTCAA
		TTGCGTATTTTCATTGATGTTG
	CYP735 A1	CCAAAGTTGGCATTTGTGAA
		GCTTGGCTAAACGTTTCCAT
